# *Polygalae Radix* shortens the circadian period through activation of the CaMKII pathway

**DOI:** 10.1080/13880209.2022.2048863

**Published:** 2022-03-17

**Authors:** Atsushi Haraguchi, Keisuke Saito, Yu Tahara, Shigenobu Shibata

**Affiliations:** School of Advanced Science and Engineering, Laboratory of Physiology and Pharmacology, Waseda University, Tokyo, Japan

**Keywords:** Crude drug, clock gene expression rhythm, locomotor activity rhythms, free-running period length

## Abstract

**Context:**

The mammalian circadian clock system regulates physiological function. Crude drugs, containing *Polygalae Radix*, and Kampō, combining multiple crude drugs, have been used to treat various diseases, but few studies have focussed on the circadian clock.

**Objective:**

We examine effective crude drugs, which cover at least one or two of Kampō, for the shortening effects on period length of clock gene expression rhythm, and reveal the mechanism of shortening effects.

**Materials and methods:**

We prepared 40 crude drugs. In the *in vitro* experiments, we used mouse embryonic fibroblasts from PERIOD2::LUCIFERASE knock-in mice (background; C57BL/6J mice) to evaluate the effect of crude drugs on the period length of core clock gene, *Per2*, expression rhythm by chronic treatment (six days) with distilled water or crude drugs (100 μg/mL). In the *in vivo* experiments, we evaluated the free-running period length of C57BL/6J mice fed AIN-93M or AIN-93M supplemented with 1% crude drug (6 weeks) that shortened the period length of the PERIOD2::LUCIFERASE expression rhythm in the *in vitro* experiments.

**Results:**

We found that *Polygalae Radix* (ED_50_: 24.01 μg/mL) had the most shortened PERIOD2::LUCIFERASE rhythm period length in 40 crude drugs and that the CaMKII pathway was involved in this effect. Moreover, long-term feeding with AIN-93M+*Polygalae Radix* slightly shortened the free-running period of the mouse locomotor activity rhythm.

**Discussion and conclusions:**

Our results indicate that *Polygalae Radix* may be regarded as a new therapy for circadian rhythm disorder and that the CaMKII pathway may be regarded as a target pathway for circadian rhythm disorders.

## Introduction

The circadian clock is genetically preserved in almost all species. In mammals, the circadian clock is composed of several clock genes, such as *cryptochrome1/2* (*Cry1/2*), *period1/2* (*Per1/2*), *brain* and *muscle arnt-like 1* (*Bmal1*) and *circadian locomotor output cycles kaput* (*Clock*) (Buhr and Takahashi 2013). These clock genes produce a rhythm of approximately 24 h through the formation of a transcriptional and translational feedback loop (Takahashi 2017). The circadian clock is present in almost all organs, and a central clock, located in the hypothalamus, orchestrates peripheral clocks located in peripheral organs. The mammalian circadian clock plays an important role in maintaining physiological functions, such as metabolism, body temperature and sleep–wake behaviour (Gachon et al. 2004). Actually, deletions or mutations in clock genes result in the dysfunction of the metabolic system and arrhythmicity of food intake (Marcheva et al. 2010; Eckel-Mahan et al. 2012; Fustin et al. 2012). Therefore, maintaining a normal circadian clock is important for maintaining normal physiological functions.

Various nutrients are known to affect the circadian clock system. For example, consumption of carbohydrate-rich foods can induce a phase shift in the liver (Hirao et al. 2009; Furutani et al. 2015). In addition, there are many reports indicating that caffeine alters the circadian clock of a variety of organisms, including humans (Oike et al. 2011; Narishige et al. 2014; Burke et al. 2015). Caffeine prolongs the free-running period of locomotor activity in mice (Oike et al. 2011) and alters the phase of peripheral clocks (Narishige et al. 2014). The above studies were mostly aimed at investigating the rhythm period prolongation of clock gene expression or altering the rhythm phase of clock gene expression. However, we thought it is important to investigate substances that shorten the circadian clock period as many humans show longer endogenous circadian periods than 24 h (Brown et al. 2008). In addition, mammals, including mice and humans, find it more difficult to adjust to a short period length than a long period length as delaying the onset of activity rhythms is easier than accelerating them (Herichová et al. 2014; West et al. 2017). Therefore, in this study, we focussed on crude drug candidates capable of shortening clock gene expression rhythms.

In this study, we focussed on evaluating the effects of crude drugs. Crude drugs and Kampo, produced by combining multiple crude drugs, are commonly used by Chinese and Japanese populations (Ikegami et al. 2006) to treat a variety of ailments (Jiang et al. 2010; Yan et al. 2014). Particularly, Kampō preparations are used as general OTC and prescription drugs in Japan. Kampō (138 different patterns) is considered safe because it is usually composed of 5–9 out of the 114 crude drugs used in Japan, most of which are prepared from safe herbs. Moreover, our previous study showed that crude drugs may act as regulators of peripheral circadian clock phases, including those of the liver and kidney (Motohashi et al. 2017).

In this study, 40 crude drugs that are used in the formulation of at least one or two Kampō preparations, and that were evaluated in a previous study (Motohashi et al. 2017), were prepared. This study screens crude drugs that have potential shortening effects on the clock gene expression rhythm in mouse embryonic fibroblasts (MEFs) and on the free-running period of mice. In addition, we determine the mechanism by which they elicit this shortening effect.

## Materials and methods

### Measurement of bioluminescence in PERIOD2::LUCIFERASE (PER2::LUC) MEFs

The rhythmic expression of PER2::LUC was measured using a real-time LUC assay in MEFs derived from PER2::LUC knock-in mice (background; C57BL/6J mice) (Yoo et al. 2004). The MEFs were cultured in Dulbecco’s modified Eagle medium (D-MEM; Fujifilm, Tokyo, Japan), supplemented with heat-inactivated 10% foetal bovine serum (FBS; Bio West, Riverside, MO) and 1% penicillin/streptomycin (Fujifilm, Tokyo, Japan) and were passaged twice a week. MEFs were incubated at 37 °C in a 5% CO_2_ incubator. MEFs were passaged at a concentration of 6 × 10^4^ cells onto 35-mm dishes (AGC Techno Glass Co. Ltd., Shizuoka, Japan). The following day, MEFs were stimulated with 100 nM dexamethasone (Sigma-Aldrich, St. Louis, MO) for 2 h to synchronize the clock gene expression rhythm before being placed in D-MEM (Wako, Osaka, Japan) supplemented with 0.1 mM d-luciferin sodium salt (Invitrogen, Carlsbad, CA) and 10% heat-inactivated FBS. The MEFs were incubated at 37 °C, and bioluminescence was monitored for 1 min at 10 min intervals for six days using a dish-type luminometer (LumiCycle 32, Actimetrics, Wilmette, IL).

### Screening for crude drugs affecting period length and circadian amplitude

In experiment 1, each crude drug was added to the culture medium at a final concentration of 100 μg/mL before bioluminescence measurements were taken. The crude drugs are listed in Table 1. Kampō is usually composed of several crude drugs, and we listed 40 crude drugs that cover at least one or two of the 138 different Kampō preparations. All crude drugs were obtained from Tsumura Co., Ltd. (Tokyo, Japan).

**Table 1.** List of crude drugs and their proportions in 138 Kampō preparations.

Number	Crude drug name	Latin name	% in 138 Kampō
1	Astragalus root	*Astragali Radix*	10.1
2	Polygalae root	*Polygalae Radix*	2.2
3	Glycyrrhiza	*Glycyrrhizae Radix*	67.4
4	Bupleurum root	*Bupleuri Radix*	15.9
5	Gardenia fruit	*Gardeniae Fructus*	8.7
6	Fresh ginger rhizome	*Zingiberis Rhizoma*	8.7
7	Cnidium rhizome	*Cnidii Rhizoma*	18.1
8	Atractylodes Lancea rhizome	*Atractylodis Lanceae Rhizoma*	24.6
9	Citrus unshiu peel	*Aurantii Nobilis Pericarpium*	17.4
10	Angelica root	*Angelicae Radix*	26.8
11	Scutellaria root	*Scutellariae Radix*	19.6
12	Cinnamon bark	*Cinnamomi Cortex*	28.3
13	Ginseng	*Ginseng Radix*	26.8
14	Pinellia rhizome	*Pinelliae Rhizoma*	19.6
15	Atractylodes rhizome	*Atractylodis Rhizoma*	5.8
16	Tuckahoe mushroom	*Poria*	33.3
17	Rehmannia root	*Rehmanniae Radix*	15.9
18	Peony root	*Paeoniae Radix*	31.9
19	Rhubarb rhizome	*Rhei Rhizoma*	11.6
20	Ephedra herb	*Ephedrae Herba*	9.4
21	Coptis rhizome	*Coptidis Rhizoma*	8.0
22	Dried ginger rhizome	*Zingiberis Siccatum Rhizoma*	37.0
23	Notopterygium rhizome	*Notopterygii Rhizoma*	2.2
24	Apricot kernel	*Armeniacae Semen*	6.5
25	Achyranthes root	*Achyranthis Radix*	2.2
26	Schisandra fruit	*Schisandrae Fruits*	3.6
27	Cornus fruit	*Corni Fructus*	2.2
28	Zanthoxylum fruit	*Zanthoxyli Fructus*	28.3
29	Dioscorea rhizome	*Dioscoreae Rhizoma*	2.9
30	Plantain seed	*Plantaginis Semen*	2.9
31	Alisma rhizome	*Alismatis Rhizoma*	10.1
32	Polyporus sclerotium	*Polyporus*	4.4
33	Ophiopogon tuber	*Ophiopogonis Tuber*	8.0
34	Mentha herb	*Menthae Herba*	5.1
35	Aconite root	*Aconiti Radix*	4.4
36	Saposhnikovia root	*Saposhnikoviae Radix*	8.0
37	Peony root	*Paeoniae Radix*	5.8
38	Platycodon root	*Platycodi Radix*	8.7
39	Immature orange	*Aurantii Fructus Immaturus*	10.1
40	Magnolia bark	*Magnoliae Cortex*	8.7

### Cell count

We collected all cells from each dish after culturing them in each medium for six days. Viable cells were counted by Trypan blue dye exclusion (Wako, Osaka, Japan). The cell count was determined using a TC10TM automated cell counter (Bio-Rad, Hercules, CA).

### Screening for signal pathways affecting PER2::LUC rhythm period length

In experiment 5, a CaMKII inhibitor (KN93 10 μM; Wako, Osaka, Japan), an inactive analogue of KN93 (KN92; 10 μM; Sigma-Aldrich, St. Louis, MO), or an ERK 1/2 inhibitor (U0126; 10 μM; Wako, Osaka, Japan) was added to the culture medium before bioluminescence measurements were taken.

### Animals

Eight-week-old male C57BL/6J mice were housed in an animal room maintained at a temperature of 22 ± 2 °C, humidity of 60 ± 5%, and a 12 h light/dark cycle (lights on from 08:00 to 20:00; light–dark (LD) condition). Zeitgeber time (ZT) 0 was defined as the lights-on time, and ZT12 was defined as the lights-off time. The mice were provided a regular diet (EF; Oriental Yeast Co. Ltd., Itabashi, Japan) and water *ad libitum* before the experiments. Experimental animal care was conducted with the authorization of the Animal Welfare Committee of Waseda University (2015-A37).

### Diets

Mice had been fed with AIN-93M before the *in vivo* experiment. We fed mice with AIN-93M supplemented with or without crude drug, which had the most shortening effect on the period length in the *in vitro* experiments. This crude drug was given for 2 weeks under LD condition and then for 4 weeks under constant dark (DD) condition. We evaluated the locomotor activity levels under LD and DD conditions, and calculated the free-running period length, which is regarded as an endogenous rhythm in the absence of environmental light signals, under DD condition. In the previous experiments, *in vivo* administration dose of herbal medicines widely ranged from 0.5 g/kg (Motohashi et al. 2017) to 1 g/kg (Ito et al. 2022) and 3–5 g/kg (Zhou et al. 2020). Based on these previous experiments, we decided to use 1% containing food (approximately 1 g/kg per day).

### Locomotor activity rhythm analysis

Locomotor activity was monitored using an SE-10 infra-red radiation sensor (Akizuki Denshi Tsusho Co. Ltd., Saitama, Japan) and analysed using the CLOCKLAB software (Actimetrics, Wilmette, IL), as previously described (Haraguchi et al. 2018). In the CLOCKLAB software, the free-running period length was calculated in mice kept under DD condition using the chi-squared periodogram (Sokolove and Bushell 1978). When mice were kept under DD condition, activity offset time is defined as the circadian time (CT) 0 and activity onset time as CT12, and CT0–12 was regarded as subject inactive (light) period and CT12–24 as subject active (dark) period. To draw graphs for locomotor activity rhythm, we used averaged data for 2 week (LD condition) and 4 weeks (DD condition), and original data (one-minute bins) were smoothed using an adjusting-averaging method with 1 h running means.

### Evaluation of clock gene expression rhythms in MEFs

**Figure 1.**
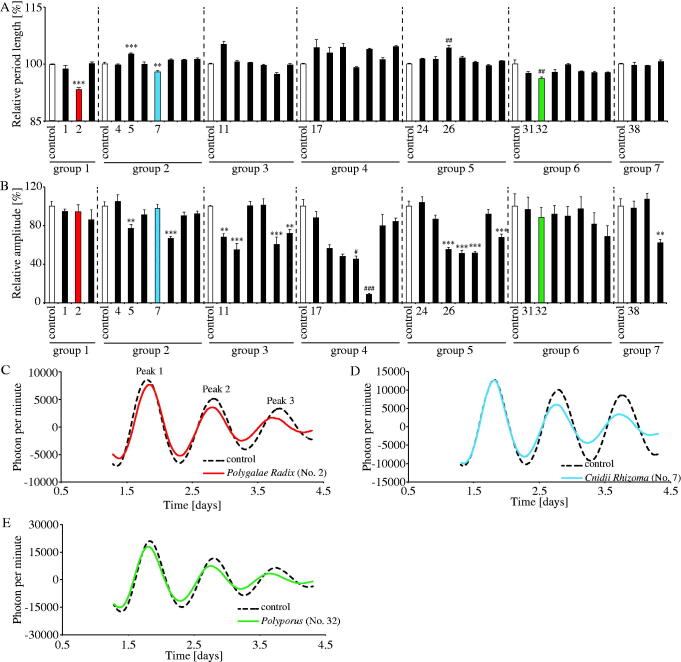
Effects of crude drugs on PER2::LUC expression rhythms. (A,B) Effects of crude drugs on rhythmic PER2::LUC expression period length and PER2::LUC bioluminescence amplitude of peak 1 in MEFs. The relative period length and amplitude of control in each group was set to 100%. The numbers correspond to each crude drug as shown in Table 1. (C–E) Representative de-trended data for MEFs treated with distilled water (control), *Polygalae Radix*, *Cnidii Rhizoma* and *Polyporus*. Data are presented as mean ± SEM (*n* = 4). **p < 0.01, ***p < 0.001 versus control in each group (one-way ANOVA with Dunnett’s multiple comparisons test). ^#^*p* < 0.05, ^##^*p* < 0.01, ^###^*p* < 0.001 versus control in each group (Kruskal–Wallis test with Dunn’s multiple comparisons test).

Original data (1-min bins) were smoothed using an adjusting-averaging method with 2 h running means, as previously described (Hayasaka et al. 2007; Ohta et al. 2008). Then, the data set was de-trended by subtracting the 24 h running average from the raw data using the R software (R Development Core Team (R Foundation for Statistical Computing, Vienna, Austria); http://www.r-project.org/). Peaks were defined as points at which the bioluminescence was higher than both sides and were confirmed from the waveform. Bioluminescence was measured for 6–10 consecutive days. We numbered them in order starting from the front (Figure 1(C)). The amplitude was calculated as the PER2::LUC bioluminescence of peak 1, and the relative amplitude of control was set to 100%. To measure bioluminescence in MEFs, the period length was calculated as half the interval between peaks 1 and 3 of the waveform.

### Data analysis

All values are expressed as the mean ± standard error of the mean (SEM). Statistical analysis was performed using GraphPad Prism version 6.03 (GraphPad Software, La Jolla, CA). We verified whether data were normally or non-normally distributed using the D’Agostino–Pearson normality test/one-sample *t*-test and whether they showed equal or biased variation using the *F*-value test/Bartlett’s test. Parametric analysis was performed using one-way analysis of variance (ANOVA) with Tukey’s or Dunnett’s multiple comparisons test, or two-way ANOVA with Tukey’s multiple comparisons test, while non-parametric analysis was conducted using the Mann–Whitney test, the Kruskal–Wallis test with Dunn’s multiple comparisons test, or the Mann–Whitney test with false discovery rate (FDR) multiple testing correction. In addition, GraphPad Prism (GraphPad Software, La Jolla, CA) calculated the ED_50_ and 95% confidence intervals.

## Results

### Experiment 1: few crude drugs shortened PER2::LUC expression rhythm period length in MEFs

PER2::LUC MEFs were treated with distilled water (control) or each crude drug (100 μg/mL), and bioluminescence was monitored for several days. In this experiment, we used distilled water as a control, because we had confirmed in previous experiments that mixing 20 μL of distilled water as vehicle with 2 mL of culture medium/35-mm dishes had no effect on the PER2::LUC expression rhythm. In addition, some previous studies also used distilled water as control (Narishige et al. 2014; Motohashi et al. 2017). Crude drugs were divided into seven groups due to the limitation of dish contents (32 dishes) in the LumiCycle equipment. Every PER2::LUC expression rhythm in seven groups is shown in Supplemental Figure 1. Since the PER2::LUC expression rhythm period and the amplitude of peak 1 in each group’s control were slightly different due to differences in cultured conditions and/or the sensitivity of the LumiCycle equipment, the results were recalculated with 100% of the PER2::LUC expression rhythm period and the amplitude of peak 1 in each group’s control (Figure 1(A,B)). Compared to the control, *Polygalae Radix* (no. 2), *Cnidii Rhizoma* (no. 7) and *Polyporus* (no. 32) significantly shortened PER2::LUC expression rhythm period, while *Gardeniae Fructus* (no. 5) and *Schisandrae Fruits* (no. 26) significantly prolonged it (Figure 1(A)). Many crude drugs had no significant effect on period length; however, approximately half of these crude drugs significantly decreased the amplitude of peak 1 (Figure 1(B)). Based on these results, we focussed on the shortening effects of *Polygalae Radix*, *Cnidii Rhizoma* and *Polyporus* in subsequent experiments, because these crude drugs did not significantly decrease the amplitude of peak 1 and a previous study identified only a few chemicals that could shorten the circadian period (Chen et al. 2012). Indeed, comparing the waveform results treated with three crude drugs which showed the shortening effects with those of control groups, the PER2::LUC peak time and amplitude of peak 1 were similar values, and the PER2::LUC peak time of peak 3 is moving forward compared to each control group, which confirmed that the PER2::LUC expression period was getting shorter (Figure 1(C–E)).

### Experiment 2: *Polygalae Radix* significantly shortened PER2::LUC expression rhythm period in MEFs

**Figure 2.**
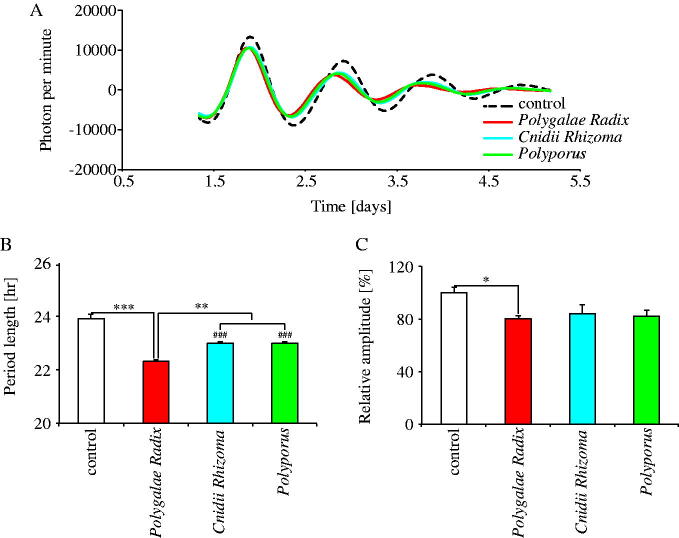
Comparison of the effects of *Polygalae Radix*, *Cnidii Rhizoma* and *Polyporus* on PER2::LUC expression rhythm in MEFs. (A) Representative de-trended data for cells treated with distilled water (control), *Polygalae Radix*, *Cnidii Rhizoma* and *Polyporus*. (B) Effect of each crude drug on rhythmic PER2::LUC expression period length. (C) Effect of each crude drug on the PER2::LUC bioluminescence amplitude of peak 1. Data are presented as mean ± SEM (*n* = 4). *p < 0.05, **p < 0.01, ***p < 0.001 (one-way ANOVA with Tukey’s multiple comparisons test). ^###^*p* < 0.001 versus control (one-way ANOVA with Tukey’s multiple comparisons test).

We compared the effects of the three crude drugs on PER2::LUC rhythm period and amplitude under the same experimental conditions (Figure 2). Comparing the waveforms results treated with each crude drug and distilled water, the three crude drugs shortened the PER2::LUC expression rhythm period (Figure 2(A)). Indeed, the three crude drugs significantly shortened period length as compared to the control group, with *Polygalae Radix* being the most effective (Figure 2(B)). Moreover, *Polygalae Radix* significantly decreased the amplitude of the peak 1 as compared to the control (*p* = 0.049) (Figure 2(C)). These results indicated that of the 40 crude drugs, *Polygalae Radix* had the most significant shortening effect on period length.

### Experiment 3: *Polygalae Radix* was not cytotoxic

**Figure 3.**
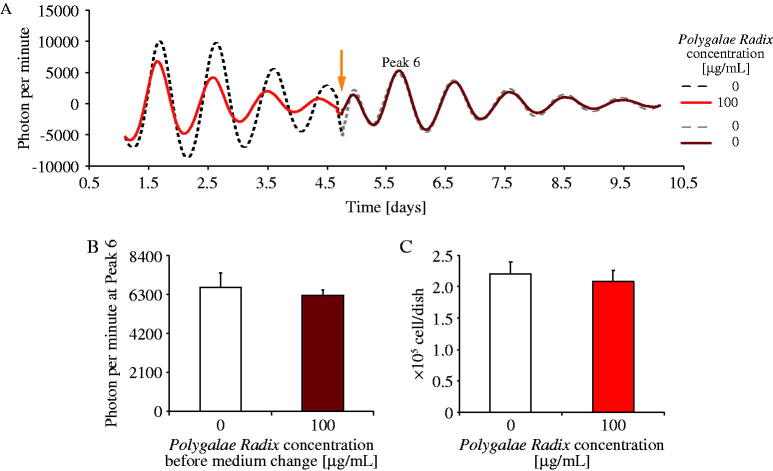
Comparison of the effects of different *Polygalae Radix* concentrations on cell survival. (A) Representative de-trended data on the effects of *Polygalae Radix* on PER2::LUC bioluminescence rhythms after medium change. The triangle indicates the time of change to a fresh regular medium. (B) PER2::LUC bioluminescence amplitude of peak 6 after changing to a fresh regular medium. Data are presented as mean ± SEM (*n* = 8). (C) MEF cell survival count after incubation under different conditions for six days. Data are presented as mean ± SEM (*n* = 4).

Experiments 1 and 2 showed that *Polygalae Radix* had the most significant shortening effect on period length. However, it also decreased PER2::LUC amplitude due to cytotoxicity or desynchronization of cellular clocks. Therefore, we conducted two separate experiments; first, we replaced the medium containing 0 or 100 μg/mL *Polygalae Radix* with a fresh medium containing 0 μg/mL *Polygalae Radix* on day 5 (arrow head), and we measured the amplitude of peak 6 (Figure 3(A,B)). The ‘100 μg/mL *Polygalae Radix*’ group was found to show a decline amplitude of each peak (peaks 1–4); however, after the change of medium, the amplitude of each peak was recovered to the similar levels as those of peaks in the control group (Figure 3(A,B)). Second, we determined MEF living cell count on day 6 after culturing in each medium (0 or 100 μg/mL *Polygalae Radix*). The number of living cells in both groups (0 or 100 μg/mL *Polygalae Radix*) was similar (Figure 3(C)). Indeed, in previous studies, cerebral cortical neurons (embryonic day 14; ddY mice) were exposed to 100 μg/mL of water extract of *Polygalae Radix* and adipocytes were exposed to 500 μg/mL extract of *Polygalae Radix* (Kuboyama et al. 2017; Wang et al. 2017). According to above previous papers, current results indicate that the cytotoxicity of 100 μg/mL *Polygalae Radix* may be weak and that low amplitude may be caused by desynchronization of PER2::LUC rhythms among cells.

### Experiment 4: *Polygalae Radix* had a dose-dependent shortening effect on PER2::LUC expression rhythm period length in MEFs

**Figure 4.**
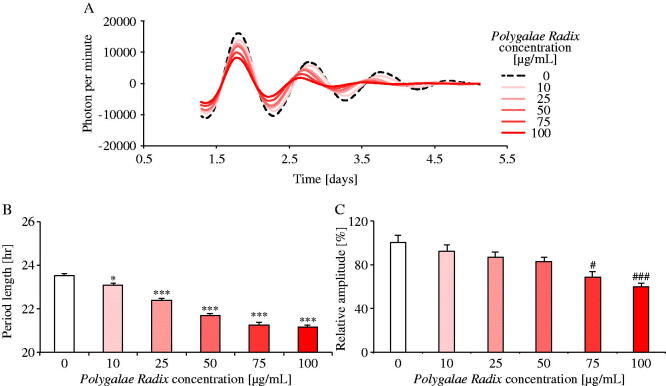
Comparison of the effects of different *Polygalae Radix* concentrations on PER2::LUC expression rhythms in MEFs. (A) Representative de-trended data for MEFs treated with different *Polygalae Radix* concentrations. (B) Effects of the different *Polygalae Radix* concentrations on rhythmic PER2::LUC expression period length. (C) Effects of the different *Polygalae Radix* concentrations on the PER2::LUC bioluminescence amplitude of peak 1. Data are presented as mean ± SEM (0 μg/mL, *n* = 6; 25 and 75 μg/mL, *n* = 4; 10, 50 and 100 μg/mL, *n* = 8). *p < 0.05, ***p < 0.001 versus 0 μg/mL (one-way ANOVA with Dunnett’s multiple comparisons test). ^#^*p* < 0.05, ^###^*p* < 0.001 versus 0 μg/mL (Kruskal–Wallis test with Dunn’s multiple comparisons test).

To evaluate the dose-dependency of *Polygalae Radix*, MEFs were treated with various concentrations of *Polygalae Radix* (0, 10, 25, 50, 75 and 100 μg/mL; Figure 4). Comparing the waveforms results treated with each dose of *Polygalae Radix* and distilled water, the PER2::LUC expression rhythm period became shorter and the amplitude of peak 1 became lower (Figure 2(A)) in response to increasing dose of *Polygalae Radix*. Indeed, it significantly shortened period length when its concentration was above 10 μg/mL in a dose-dependent manner (Figure 4(B)). Moreover, based on these results, the ED_50_ for period shortening effect was 24.01 μg/mL (95% confidence intervals: 20.71–27.82). At concentrations above 50 μg/mL, *Polygalae Radix* significantly decreased the amplitude (Figure 4(C)). In addition, we found that *Polygalae Radix* shortened *Bmal1-ELuc* rhythm periods in *Bmal1-ELuc* MEFs (Figure S2). These results indicated that *Polygalae Radix* elicited its shortening effect in a dose-dependent manner.

### Experiment 5: *Polygalae Radix* shortened PER2::LUC expression rhythm period length through the phosphorylation activity of CaMKII

**Figure 5.**
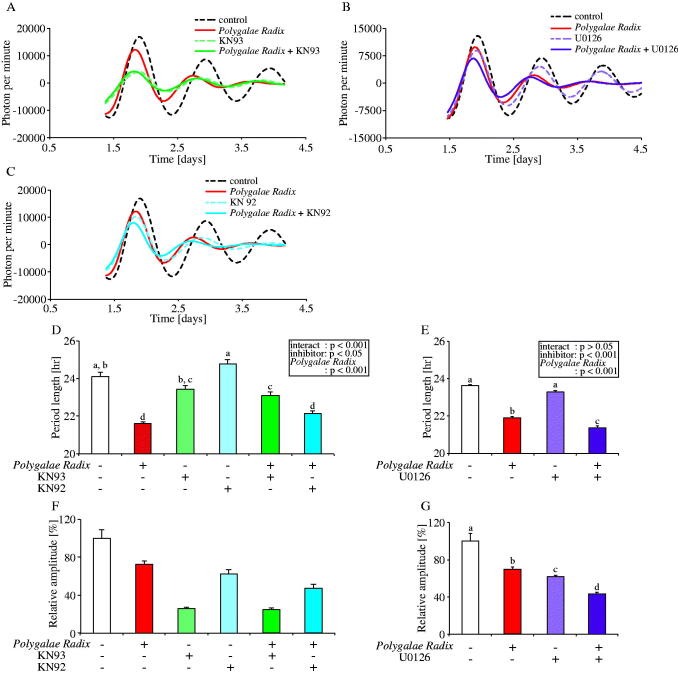
Effects of CaMKII and ERK 1/2 inhibition on the PER2::LUC expression rhythm of *Polygalae Radix*-treated MEFs. (A,C) Representative de-trended data for MEFs treated with 0.25% DMSO (control), *Polygalae Radix*, KN93 and KN92. (B) Representative de-trended data for MEFs treated with 0.25% DMSO (control), *Polygalae Radix* and U0126. (D,E) Effects of KN93, KN92 and U0126 on the rhythmic PER2::LUC expression period length of *Polygalae Radix*-treated MEFs; the two-way ANOVA results are shown. (F,G) Effects of KN93, KN92 and U0126 on the PER2::LUC bioluminescence amplitude of peak 1 in *Polygalae Radix*-treated MEFs. Data are presented as mean ± SEM (*Polygalae Radix* in E and G, U0126, and *Polygalae Radix*+U0126, *n* = 8; other groups, *n* = 4). We determined statistical values using two-way ANOVA with Tukey’s multiple comparisons test in D and E, and the Mann–Whitney test with false discovery rate (FDR) multiple testing correction in F and G. Different letters denote significant difference (*p* < 0.05).

A previous study reported that an oligosaccharide ester, originating from the roots of *Polygalae Radix*, activated the CaMKII and ERK1/2 pathways (Hu et al. 2014). To investigate the mechanism by which *Polygalae Radix* elicits its effects on PER2::LUC expression rhythm period length in MEFs, we prepared KN93 and U0126 for CaMKII and ERK1/2 inhibition, respectively (Figure 5). We also used KN92, an inactive control drug for KN93. Comparing the waveforms results treated with each condition, all inhibitor decreased the amplitude of peak 1, and only KN93 inhibited the shortening effects of *Polygalae Radix* (Figure 5(A–C)). Indeed, KN93 significantly attenuated the shortening effect of *Polygalae Radix* on period length (Figure 5(D)). However, KN92 had no effect on the period length shortening effect of *Polygalae Radix* (Figure 5(D)). Treatment with KN93 or *Polygalae Radix*+KN93 decreased amplitude (Figure 5(F)). U0126 did not affect the shortening effect of *Polygalae Radix* on period length (Figure 5(E,G)).

In addition, to identify what component of *Polygalae Radix* has the shortening effect on biological clock, we examine the effects of tenuifolin. Tenuifolin is one of the main components of *Polygalae Radix* and elicits sleep-promoting effects in zebrafish (Chen et al. 2020). However, tenuifolin had no effects on period length and amplitude of clock gene expression rhythm (Figure S3).

These results indicated that *Polygalae Radix* induced shortening of PER2::LUC expression rhythm period length through the activation of CaMKII.

### Experiment 6: *Polygalae Radix* shortened the free-running period in mice under DD conditions

**Figure 6.**
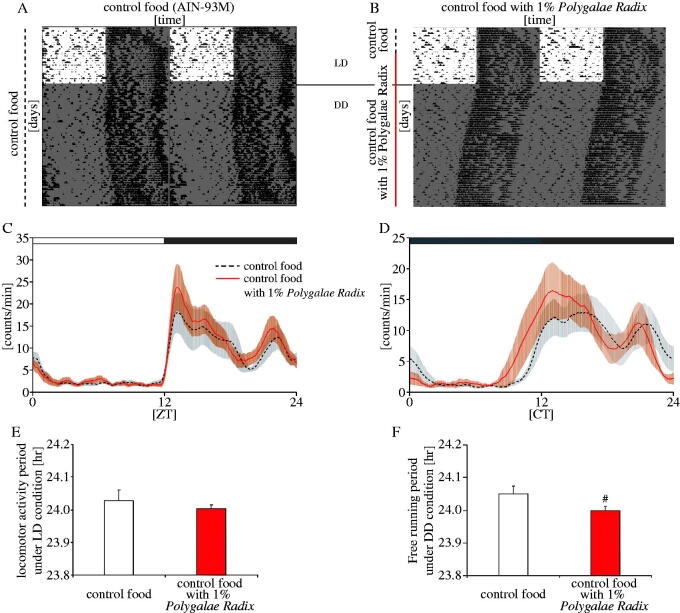
Effects of *Polygalae Radix* on the free-running period of mice. (A,B) Representative double-plotted actograms of locomotor activity in mice treated with AIN-93M or AIN-93M supplemented with 1% *Polygalae Radix*. The dark shadow indicates the dark period. The black dashed bars and red bar indicate the duration of administration of AIN-93M and AIN-93M supplemented with 1% *Polygalae Radix*, respectively. (C,D) Locomotor activity rhythms under LD or DD conditions. The open and closed bars indicate the light and dark periods, respectively. (E) Locomotor activity period in each group under LD condition. (F) Free-running period in each group under DD condition. Data are presented as mean ± SEM (*n* = 5). ^#^*p* < 0.05 versus control (Mann–Whitney’s test).

We examined the effects of *Polygalae Radix* on the free-running period of mice under DD condition as a switch from *in vitro* experiments to *in vivo* experiments. Mice were acclimated to control food (AIN-93M) for the first week, divided into the ‘control food’ and ‘*Polygalae Radix*’ groups, and fed with control food and control food supplemented with 1% *Polygalae Radix*, respectively (Figure 6(A,B)). Considering that oral administration of water extract of *Polygalae Radix* was 14.02 g/kg for LD_50_ (Guan et al. 2012) and that the other crude drugs used in our previous experiment were orally administration of 500 mg/kg (Motohashi et al. 2017), the amount of *Polygalae Radix* added to the control diet was set at 1% in the present experiment, which was the equivalent of approximately 1 g/kg. Mice were fed thus for 2 weeks under LD condition, and then released under DD condition for the following 4 weeks. Locomotor activity rhythms in the ‘1% *Polygalae Radix*’ group under LD or DD conditions were higher around ZT12 and CT12 than in the control group (Figure 6(C,D)). *Polygalae Radix* had no effect on locomotor activity rhythm period length under LD condition (Figure 6(E)). However, it significantly shortened the free-running period length under DD condition (Figure 6(F)). Collectively, these results indicated that *Polygalae Radix* shortened free-running period length in mice.

## Discussion

Several studies have identified pharmacologically active and bioactive compounds that affect clock gene expression rhythm period length *in vitro* and/or *in vivo* (Isojima et al. 2009; Yagita et al. 2009; Hirota et al. 2010). However, only a few compounds that shorten clock gene expression rhythm period length have been identified (Hirota et al. 2008; Tamai et al. 2018). In this study, we screened 40 crude drugs for period shortening effects on clock gene expression and locomotor activity rhythms. This screening revealed three crude drugs with period shortening effects, and *Polygalae Radix* was found to show the most significant effect. In addition, we found that the effects of *Polygalae Radix* were suppressed by the inhibition of CaMKII activation. Moreover, the period shortening effects of *Polygalae Radix* were confirmed *in vivo*, and included the evaluation of the free-running period of locomotor activity rhythm in mice.

The *in vitro* experiments showed that *Polygalae Radix* decreased the amplitude of PER2::LUC and *Bmal1-Eluc* expression rhythms to the same proportion as it shortened period length. Other *in vitro* experiments revealed that *Polygalae Radix* was not cytotoxic, as *Polygalae Radix* had no effect on living cell count after six days of culturing and MEFs cultured in media containing 0 or 100 μg/mL *Polygalae Radix* showed similar bioluminescence levels after medium change. As well known, during long-term cell culturing, the circadian rhythm is attenuated when each cell is out of phase. However, medium change resets the rhythm and restored higher amplitude rhythms. These results indicate that *Polygalae Radix* is not cytotoxic at this concentration. The ED_50_ for period shorting effect was 24.01 μg/mL, and this concentration may be quite low, because *Polygalae Radix* is complex of crude components.

We found that *Polygalae Radix* shortened not only PER2::LUC rhythm period length in MEFs but also the free-running period length in mice. Several signalling pathways, especially CaMKII pathways, are involved in the determination of biological rhythm period length (Kon et al. 2015; McCarthy et al. 2016). For example, KN93 (5 and 10 μM) was found to decrease *Bmal1*-*luc* expression rhythm period length (Kon et al. 2015; McCarthy et al. 2016). In addition, a previous study showed that *Polygalae Radix* activated the ERK1/2 and CaMKII pathways (Hu et al. 2014). In agreement with previous reports, we found that *Polygalae Radix* may activate CaMKII and shorten PER2::LUC expression rhythm period length. Moreover, a previous study showed that CamK2aK42R mutant mice, which has no kinase activity of CaMKII, showed a longer free-running period length as compared to wild type mice (Kon et al. 2014). Collectively, these results indicate that the activation level of CaMKII is an important factor in determining biological rhythm period length.

This study showed that *Polygalae Radix* activated the CaMKII signalling pathway and shortened clock gene expression rhythm and locomotor activity rhythm period lengths. However, an important limitation of this study is that we did not identify what component of *Polygalae Radix* has the shortening effect on biological rhythm. It is possible that *Polygalae Radix* also contains other substances that have already been reported to affect biological rhythms, such as catechin and nobiletin (Li et al. 2016; Shinozaki et al. 2017). Therefore, further investigations are required.

The *in vitro* experiments showed that low *Polygalae Radix* concentrations had no effect on PER2::LUC amplitude but slightly shortened PER2::LUC expression rhythm period. *In vivo* experiments showed that control food supplemented with 1% *Polygalae Radix* increased locomotor activity levels around ZT12 and CT12, and slightly shortened the free-running period. In the previous experiments, *in vivo* administration dose of herbal medicines widely ranged from 0.5 g/kg (Motohashi et al. 2017), 1 g/kg (Ito et al. 2022) and 3–5 g/kg (Zhou et al. 2020) depending on herbal variations and behavioural evaluation. In the current experiment, the amount of *Polygalae Radix* added to the control diet was set at 1%, and this concentration was the equivalent of approximately 1 g/kg per day. Based on present results along with previous observations, 1% is a low *Polygalae Radix* concentration and its shortening effects would have been better observed with increase in concentration to the 3–5% range, for example. However, increasing the concentration of *Polygalae Radix* may have decreased the amount of food consumed by mice, and decrease in food consumption leads to advance the locomotor activity rhythms (Mendoza et al. 2008). Therefore, further investigations are required on this in the future.

In previous studies, we found that as opposed to *Polygalae Radix*, *Polyporus* and *Bupleuri Radix* were good candidates for the effective acute manipulation of the peripheral circadian clock phase, with stimulation time-of-day dependency *in vitro* and *in vivo* (Motohashi et al. 2017). In this study, we found that *Polyporus* had a shortening effect and that *Bupleuri Radix* had no effect on circadian period length. These results indicate that the circadian period length pathway may be different from that of the circadian phase. Actually, previous studies have reported that nobiletin affects circadian period length and phase by stimulating different pathways (He et al. 2016; Shinozaki et al. 2017).

In conclusion, *Polygalae Radix* shortened clock gene expression rhythm period length via the CaMKII pathway and shortened the free-running period length. Compared to the effects of many medicinal drugs, crude drugs are generally better tolerated (Sardesai 2002). Moreover, a previous study showed that Ca^2+^-related pathways, including the CaMKII pathway, regulate sleep duration in mammals (Tatsuki et al. 2016). Therefore, we suggest that *Polygalae Radix* may be regarded as a new therapy and that the CaMKII pathway may be regarded as a target pathway, for disorders caused by delayed sleep phase syndrome, extreme eveningness and social jetlag. This is because these conditions require a phase advance and/or shortening of circadian rhythms.

## Supplementary Material

Supplemental Material
