# Supplementary material for: Polygalae Radix shortens the circadian period through activation of the CaMKII pathway
Source: Pharm Biol. 2022 Mar 17;60(1):689–98. doi: 10.1080/13880209.2022.2048863 (PMC8933028; doi:10.1080/13880209.2022.2048863)
Supplement: Supplemental Material [file IPHB_A_2048863_SM2713.pdf]

Supplementary Information

Polygalae Radix shortens the circadian period through the activation of the CaMKII pathway

Atsushi Haraguchi, Keisuke Saito, Yu Tahara, and Shigenobu Shibata*
Laboratory of Physiology and Pharmacology, School of Advanced Science and Engineering, Waseda University, Tokyo, Japan

*Corresponding author: Shigenobu Shibata
Laboratory of Physiology and Pharmacology, School of Advanced Science and Engineering, Waseda University, Shinjuku-ku, Tokyo 162-8480, Japan
Tel: +81-3-5369-7318, E-mail address: shibatas@waseda.jp

Supplemental methods

Measurement of bioluminescence in *Bmal1-Eluc* mouse embryonic fibroblasts (MEFs)

The rhythmic expression of *Bmal1* was measured using a real-time LUC assay in MEFs (GPC Laboratory Co. Ltd. Japan). MEFs were stimulated with 100 nM dexamethasone for 2 h to synchronize clock gene expression rhythm before being placed in D-MEM supplemented with 0.1 mM D-luciferin sodium salt and 10% heat-inactivated FBS. MEFs were incubated at 37°C, and bioluminescence was monitored for 1 min at 10-min intervals using a dish-type luminometer.

Tenuifolin

Tenuifolin is a major constituent of the *Polygala Radix*. Tenuifolin (0.5% MeOH) was added to the culture medium at a final concentration of 100 μg/mL before bioluminescence measurements were taken.

Data analysis

All values are expressed as mean ± SEM. Statistical analysis was performed using GraphPad Prism version 6.03 (GraphPad Software, USA). We verified data normality using the D'Agostino-Pearson normality test/one-sample t-test, and verified whether data showed equal or biased variation using the F-value test/Bartlett's test. Parametric analysis was performed using the t-test.

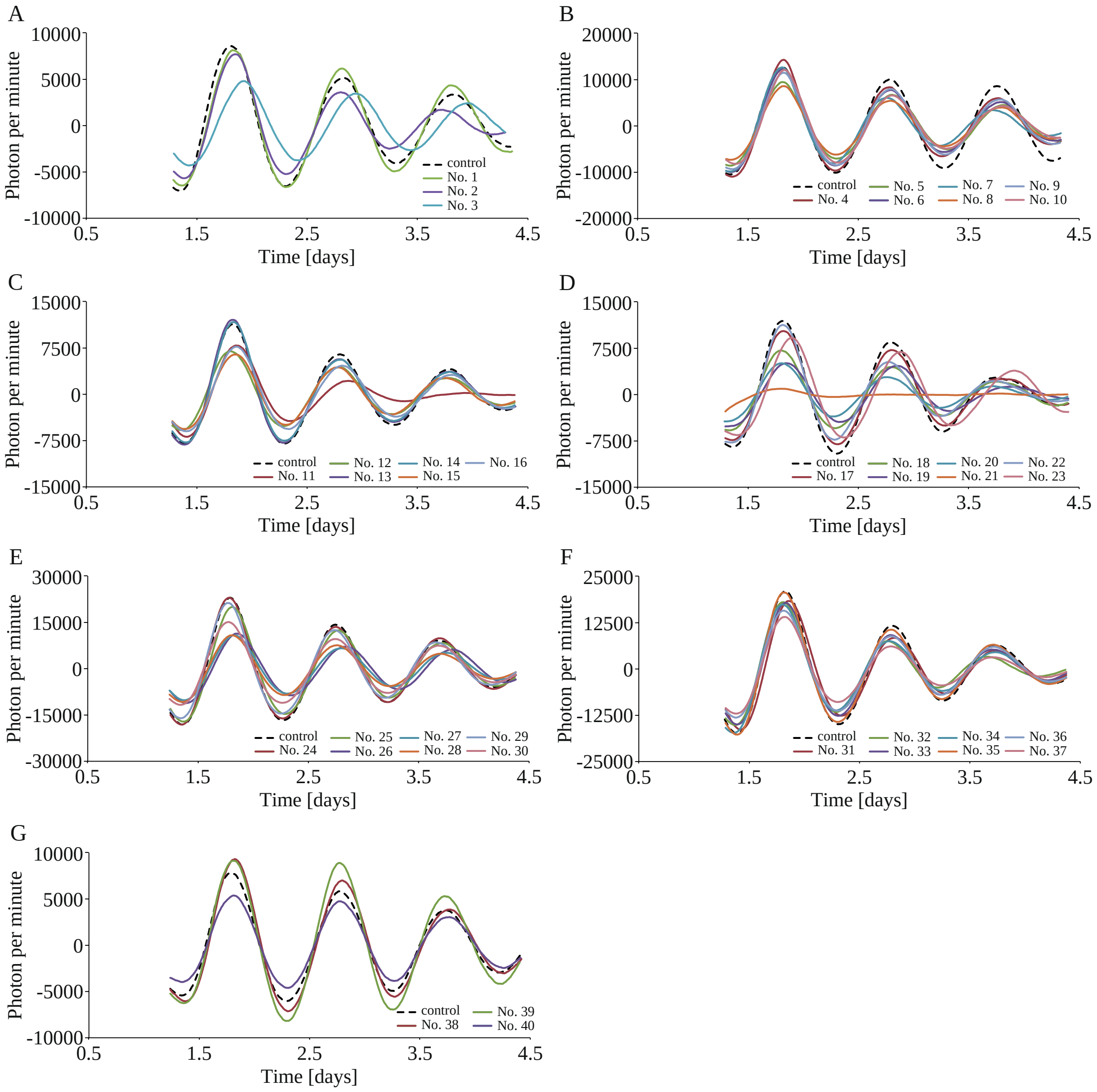

Supplementary Figure 1. Effects of crude drugs on PER2::LUC expression rhythms in MEFs. (A-G) Representative de-trended data for MEFs treated with each crude drug (100 μg/mL) in 7 groups.

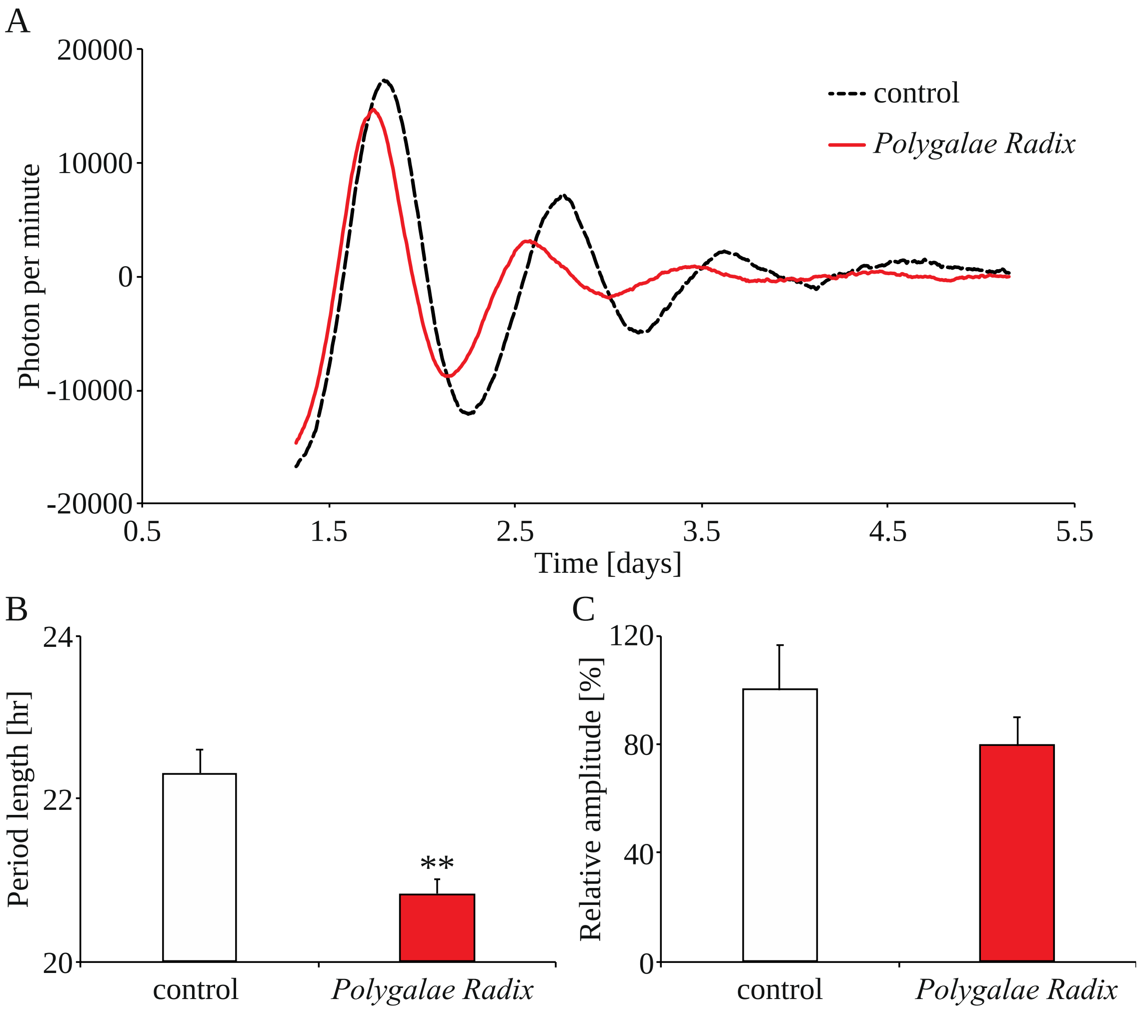

Supplementary Figure 2. Effects of *Polygalae Radix* on *Bmal1* expression rhythms in MEFs. (A) Representative de-trended data for MEFs treated with *Polygalae Radix* (100 µg/mL). (B) Effects of *Polygalae Radix* (100 µg/mL) on rhythmic *Bmal1-Eluc* expression period length. (C) Effects of *Polygalae Radix* (100 µg/mL) on the *Bmal1-Eluc* bioluminescence amplitude of peak 1. Data are presented as mean ± SEM (n = 4). ** $p < 0.01$ (t-test).

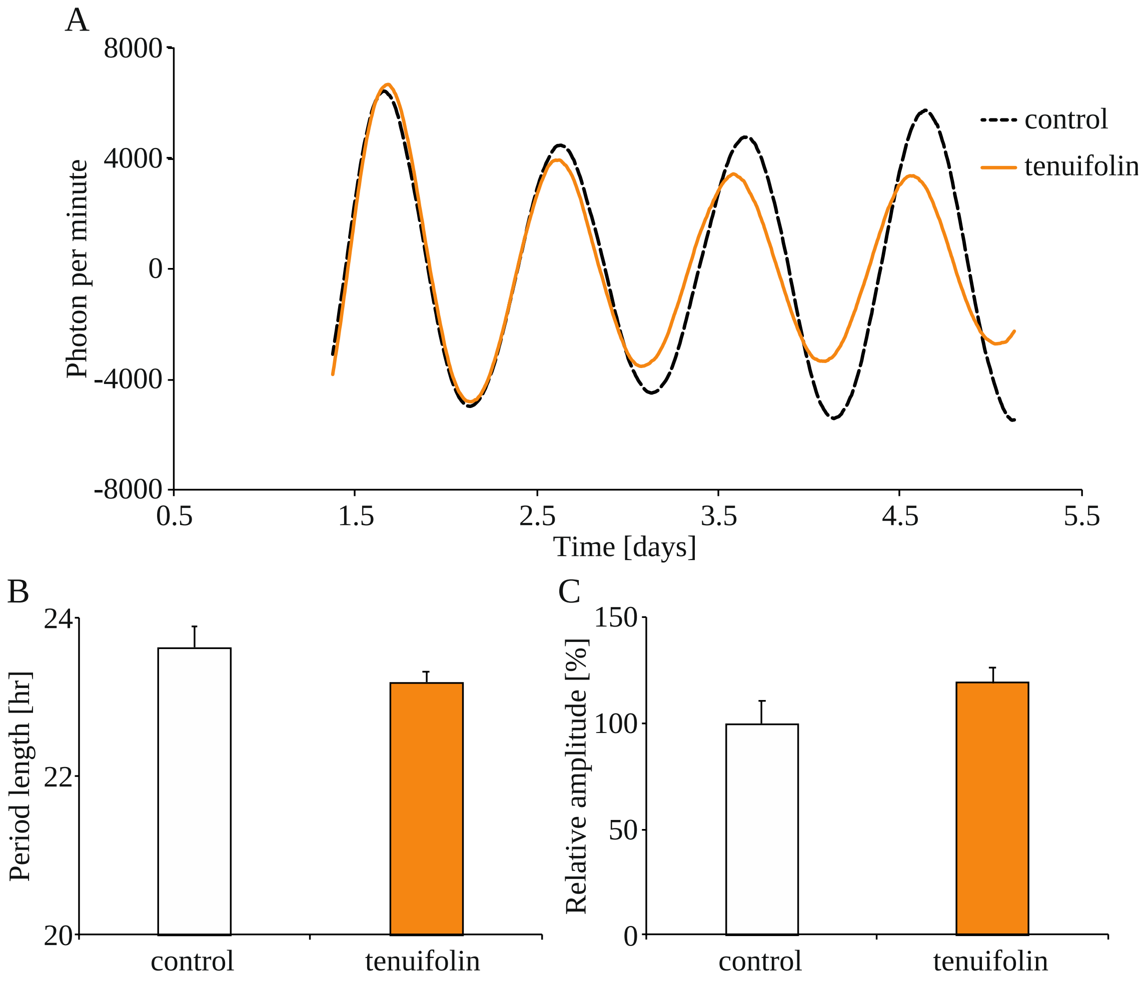

Supplementary Figure 3. Effects of tenuifolin on PER2::LUC expression rhythms in MEFs. (A) Representative de-trended data for MEFs treated with tenuifolin (100 µg/mL). (B) Effects of tenuifolin (100 µg/mL) on rhythmic PER2::LUC expression period length. (C) Effects of tenuifolin (100 µg/mL) on the PER2::LUC bioluminescence amplitude of peak 1. The control group was treated with 0.5% MeOH. Data are presented as mean ± SEM (n = 4).
